# Mechanically Reinforced Silica Aerogels via Thermally Induced Phase Separation of Poly(ethylene-co-vinyl Alcohol)

**DOI:** 10.3390/gels11110870

**Published:** 2025-10-30

**Authors:** Hainan Ma, Baomin Wang, Yongjun Zhang, Liquan Zheng

**Affiliations:** 1 College of Harbour and Coastal Engineering, Jimei University, Xiamen 361021, China; mahainanhelen@jmu.edu.cn (H.M.);; 2 Xiamen Key Laboratory of Green and Smart Coastal Engineering, Xiamen 361021, China; 3 School of Civil Engineering, Dalian University of Technology, Dalian 116024, China

**Keywords:** silica aerogels, poly(ethylene-co-vinyl alcohol), thermally induced phase separation, porosity, mechanical properties

## Abstract

Silica aerogels are highly attractive due to their outstanding properties, including their low density, ultralow thermal conductivity, large porosity, high optical transparency, and strong sorption activity. However, their inherent brittleness has limited widespread applications. Constructing a robust, highly porous three-dimensional network is critical to achieving the desired mechanical properties in aerogels. In this study, we introduce a novel synthesis route for fabricating lightweight and mechanically strong aerogels by incorporating poly(ethylene-co-vinyl alcohol) (EVOH) through thermally induced phase separation (TIPS). EVOH exhibits upper critical solution temperature (UCST) behavior in a mixture of isopropanol (IPA) and water, which can be utilized to reinforce the silica skeletal structure. Robust aerogels were prepared via the sol–gel process and TIPS method, followed by supercritical CO_2_ drying, yielding samples with bulk densities ranging from 0.136 to 0.200 g/cm^3^. N_2_ physisorption analysis revealed a mesoporous structure, with the specific surface area decreasing from 874 to 401 m^2^/g as EVOH content increased from 0 to 80 mg/mL. The introduced EVOH significantly enhanced mechanical performance, raising the flexural strength and compressive strength to 0.545 MPa and 18.37 MPa, respectively—far exceeding those of pure silica aerogel (0.098 MPa and 0.74 MPa). This work demonstrates the effectiveness of the TIPS strategy for developing high-strength, low-density silica aerogels with well-preserved porosity.

## 1. Introduction

Silica aerogels represent a distinctive class of mesoporous materials characterized by low effective density, low thermal conductivity, low refractive indices, large specific surface area, and superior porosities [1,2,3], making them promising for applications in fields such as sorption media [4,5], thermal insulators [6,7], chemical engineering [8,9], environmental remediation [10,11], drug delivery systems [12,13], sensing technologies [14,15], microelectronics [16,17], aerospace engineering [18,19], etc. However, despite their wide application range, the extensive use of silica aerogels remains significantly limited by their inherent fragility and poor mechanical strength, which hinders their processing and handling—especially in the applications of complex physical structures [20,21,22]. To overcome these inherent mechanical limitations, a wide variety of reinforcement strategies have been explored over the past two decades.

Early approaches, such as Ostwald ripening at high pH, redistributed silica mass to strengthen neck regions [23,24], while pre-condensation of silane precursors produces high molecular weight poly(diethoxysiloxane) that improves the skeletal network [25,26]. Another well-known strategy for developing mechanically strengthened aerogels is the incorporation of reinforcement materials, including fibers [27,28], carbon nanotubes [29], nanoparticles [30,31], and graphene [32]. While minimal attention has been paid to the influence of interfacial bonding on the mechanical properties of reinforced aerogels, more recent efforts have introduced organic–inorganic hybrid systems. Among them, polymer modification has attracted particular attention because it not only reinforces the silica skeleton but also imparts additional functionalities. Surface modification or crosslinking with polymers such as polyurea [33], polyurethane [34], epoxies [35], polystyrene [36], and polymethacrylates [37] and even chemical vapor deposition of cyanoacrylates or silanes [38] have been reported to enhance mechanical properties successfully. Although these methods can substantially improve mechanical robustness, the significant increase in bulk density cannot be avoided. The limitations lay in the inhomogeneous interactions and deficient reactions between the silica skeleton and polymers. Therefore, strengthening silica aerogels using various strategies without obviously increasing density and realizing production on an industrial scale at a low cost continues to be a significant challenge.

Among the various strategies, thermally induced phase separation (TIPS) has emerged as a promising approach for fabricating lightweight porous composites [39]. It has been employed to produce polystyrene gels in which tetraethoxysilane (TEOS) was polymerized to form composite aerogels [40]. TIPS can also proceed through heterogeneous nucleation by particles suspended in the cooling solution, resulting in continuous polymer coatings on the dispersed particles [41]. In our previous work, we employed a TIPS-based method to deposit poly(methyl methacrylate) (PMMA) onto silica networks, successfully obtaining strong PMMA-modified silica aerogels [42].

Herein, thermally induced phase separation of EVOH from an isopropanol/water mixture was employed to reinforce the skeletal network of silica aerogels. EVOH is a crystalline copolymer consisting of hydrophilic vinyl alcohol and hydrophobic ethylene segments, which has attracted much attention as a biomedical material owing to its hydrophilicity, biocompatibility, thermal stability, and chemical resistance [43]. Due to the upper critical solution temperature (UCST) behavior exhibited in isopropanol/water solutions, EVOH precipitated onto the silica particle surfaces during cooling [44]. The resulting aerogels maintain a homogeneous mesoporous structure while exhibiting a substantial enhancement in mechanical performance. The effects of EVOH incorporation on pore morphology, specific surface area, pore size, mechanical properties, and thermal insulation performance were systematically investigated. Notably, no additional catalyst was required during synthesis, as EVOH was directly deposited onto the silica skeleton upon cooling. The suggested TIPS strategy is advantageous due to the simple synthetic procedure and uncomplicated modification mechanism, as well as being relatively environmentally friendly compared to conventional polymer modification routes. Consequently, the TIPS-based strategy to prepare EVOH-modified silica aerogels demonstrates high efficiency in improving mechanical strength while preserving intrinsic porous features, showing promise for applications in multi-functional building materials.

## 2. Results and Discussion

### 2.1. Formation of EVOH-Modified Aerogels and Basic Properties

**Figure 1:**
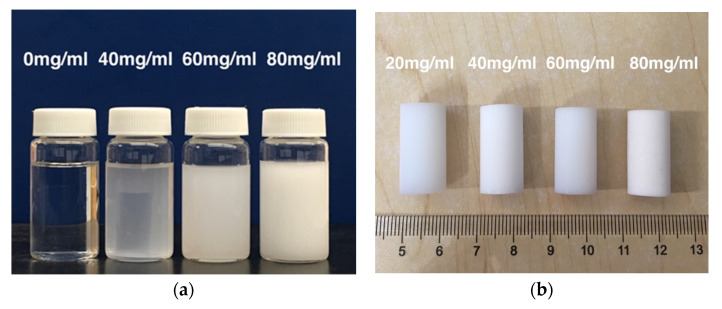
(**a**) Silica gels in suspensions of precipitated EVOH after TIPS and (**b**) EVOH-modified aerogels with different concentrations of EVOH.

The silica alcogels were synthesized through conventional two-step acid–base catalysis of TEOS, yielding transparent gels with a faint blue tint [45,46]. After sufficient aging, the gels were immersed in EVOH solutions at 70 °C for 24 h to ensure complete infiltration of the polymer into the porous network. Due to the upper critical solution temperature (UCST) behavior exhibited in isopropanol/water solutions, EVOH undergoes phase separation and precipitates onto the silica particle surfaces during the cooling process. The silica gels displayed opalescence at 10 °C, as shown in Figure 1a. Following supercritical CO_2_ drying, the resulting aerogels exhibited progressively higher whiteness and opacity with increasing EVOH concentrations used in the TIPS process, as illustrated in Figure 1b.

Beyond the increased opacity, the concentration of EVOH significantly influences the density, porosity, and linear shrinkage of the resulting aerogels. As summarized in Table 1, higher polymer concentration led to a moderate increase in bulk density and a corresponding reduction in porosity and linear shrinkage. For instance, the EA-80 aerogels exhibited an increase in bulk density to 0.200 g/cm^3^, a decrease in porosity by 6.9%, and a reduction in linear shrinkage from 8.6% to 6.9% compared to the unmodified aerogel. Notably, when compared with composite aerogels engineered through fiber doping [20,47] or polymer cross-linking [35,36,48], the EVOH-modified aerogels prepared via the TIPS method demonstrate superior retention of key properties, such as low density and high porosity.

### 2.2. Microstructural Characterization

**Figure 2:**
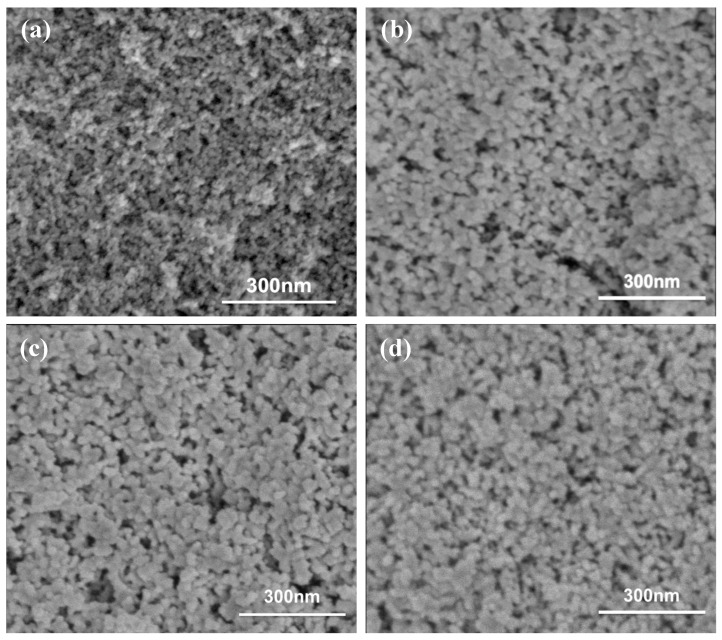
The SEM micrographs of the unmodified aerogel and EVOH-modified aerogels. (**a**) is pure silica aerogel, ρ = 0.136 g/cm^3^, (**b**) is EVOH-40, ρ = 0.181 g/cm^3^, (**c**) is EVOH-60, ρ = 0.193 g/cm^3^, and (**d**) is EVOH-80, ρ = 0.200 g/cm^3^.

The microstructural morphology of aerogels modified with varying concentrations of EVOH, as synthesized using the TIPS method, is shown in Figure 2. Scanning electron microscopy (SEM) images reveal that all EVOH-modified aerogels exhibit typical three-dimensional porous network structures with uniformly sized silica particles. In contrast, the unmodified aerogels display a more open network structure with higher porosity (93.7%) and smaller skeletal particle sizes, approximately estimated to be around 10 nm via SEM, corresponding to a lower bulk density of about 0.136 g/cm^3^. For the EVOH-modified aerogels, the skeleton particles appear larger, approximately in the range of 20–30 nm. The increase in EVOH concentration leads to a thickened network skeleton and a reduction in porosity by about 6.9%. Accordingly, the bulk density of the modified aerogel at an EVOH concentration of 80 mg/mL reaches about 0.200 g/cm^3^. The neck structures interconnecting the particles are typically the most mechanically vulnerable regions within the aerogel architecture. The precipitation of EVOH via the TIPS method wraps the silica particles and strengthens the weak junctions. Consequently, the modified aerogel network exhibits greater resistance to tensile stresses during the drying process, thus minimizing shrinkage while simultaneously enhancing compressive and flexural strength.

**Figure 3:**
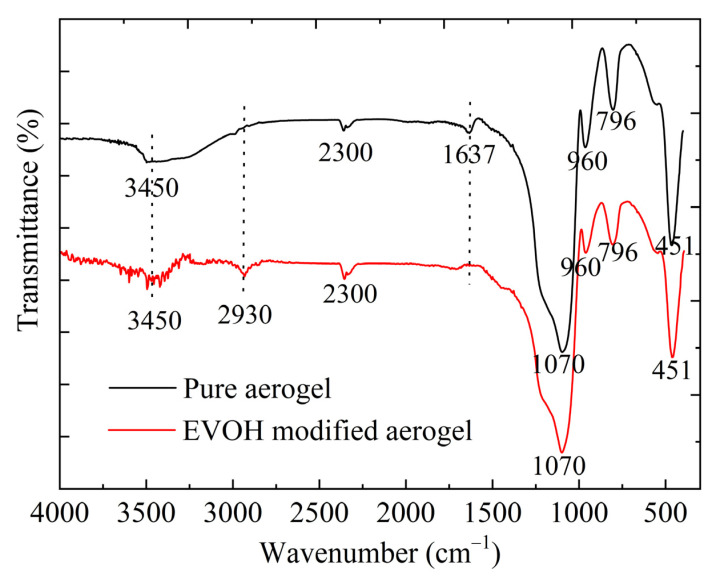
The FT-IR spectra of the pure silica aerogel and EVOH-modified aerogel.

Figure 3 presents the infrared spectra of the pure aerogel and EVOH-modified aerogel. The characteristic peaks observed at approximately 1070 cm^−1^, 796 cm^−1^, and 451 cm^−1^ are attributed to the asymmetric stretching, symmetric stretching, and bending vibrations of Si-O-Si, respectively [49], confirming that these functional groups constitute the structural network backbone of the silica aerogels. The spectrum of pure silica aerogel shows no absorption peaks at 2930 cm^−1^, indicating the absence of C-H stretching vibration and suggesting that the precursor TEOS was completely hydrolyzed during the two-step acid-base catalyzed sol–gel process. Upon the addition of EVOH, the modified aerogels exhibit characteristic peaks at 3450 cm^−1^ and 2930 cm^−1^, corresponding to the O-H stretching vibration and the C-H stretching vibration in methylene groups, respectively. These observations confirm that EVOH has been effectively deposited on the surface of the aerogel framework. Furthermore, the Si-OH stretching vibration absorption peak at 960 cm^−1^ is significantly diminished in comparison to the unmodified aerogel.

### 2.3. Pore Structure Analysis

**Figure 4:**
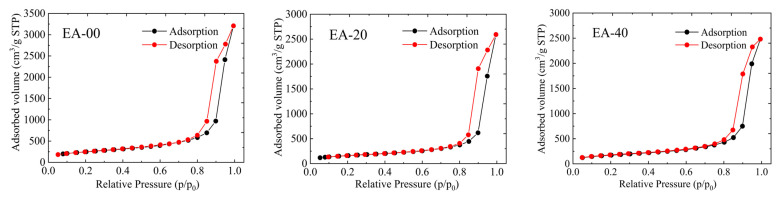

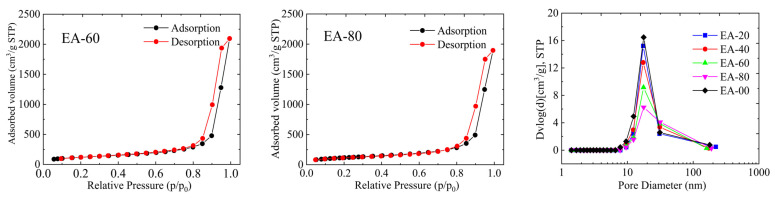
The N_2_ adsorption–desorption isotherms and pore size distribution of the EVOH-modified aerogel.

To further investigate the pore characteristics of the obtained EVOH-modified aerogels, N_2_ adsorption–desorption isotherms of the samples were measured. As illustrated in Figure 4, the aerogels exhibit an IUPAC type-IV isotherm with a distinct capillary condensation step at the relative pressure (P/P_0_) range of 0.8–1.0, demonstrating the mesoporous three-dimensional network structure that is composed of aggregated nanoparticles [50,51]. The Barrett–Joyner–Halenda (BJH) pore size distributions [52] reveal that the most probable pore size of the EVOH-modified aerogels is approximately 17–18 nm, further indicating the mesoporous nature. Additionally, the pore size distribution broadens progressively with increasing concentrations of EVOH. This result is consistent with the microstructure conducted via scanning electron microscopy (SEM), where increased EVOH concentration leads to a denser aerogel network structure with reduced pore volume and the emergence of larger pores.

The specific surface areas, pore volumes, and average pore sizes of the silica aerogels and the EVOH-modified aerogels are given in Table 2. The pure silica aerogel has a BET surface area of 871 m^2^/g and a total pore volume of 5.055 cm^3^/g. In comparison, the BET surface area and total pore volume of the EVOH-modified aerogels are smaller than those of the pure aerogel due to the deposition of EVOH on the silica skeletons. Although the specific surface area of the EA-80 sample decreased by 54%, it is still less than that reported for silica aerogels reinforced via surface modification or crosslinking with organic polymers, where surface areas decrease by 75% or more [35,36,48,53,54]. This relatively smaller reduction can be attributed to the precipitation behavior of EVOH during the TIPS process. Unlike cross-linking reactions that may generate new polymer networks within the pores, the deposition of EVOH via the TIPS method preserves a relatively uniform nanoporous structure. By adjusting the concentration of EVOH solutions, deposition can be controlled to strengthen the skeleton while minimizing excessive coverage. As a result, the mechanical properties of the aerogels are enhanced while maintaining a high porosity.

**Figure 5:**
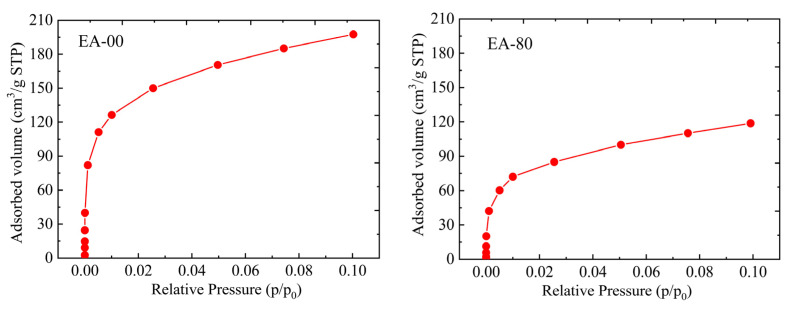
N_2_ adsorption isotherms of aerogels at low relative pressure (P/P_0_ < 0.1).

Due to the presence of both mesoporous and microporous structures in aerogels, micropores, with diameters less than 2 nm, significantly influence the specific surface area of aerogel materials [55]. In the synthesis of EVOH-modified silica aerogels via the TIPS method, the polymer not only infiltrates the mesopores but also fills some of the micropores, resulting in a reduction in specific surface area and pore volume and an increase in average pore diameter. This is evident from the comparison of the adsorption capacity at the very-low-pressure region (P/P_0_ < 0.1) between EA-00 and EA-80 (Figure 5), where the adsorption quantity significantly decreases due to the filling of micropores in the EVOH-modified aerogels. Furthermore, the reductions in specific surface area and pore volume, calculated using the Nonlocal Density Functional Theory (NLDFT) method [56,57,58] and Dubinin–Radushkevich (DR) equation [59], as shown in Table 2, further corroborate this observation. Although the filling of micropores in the modified aerogels diminishes the specific surface area, it concurrently strengthens the most fragile skeletal structures of the aerogel framework.

### 2.4. Thermal Properties

**Figure 6:**
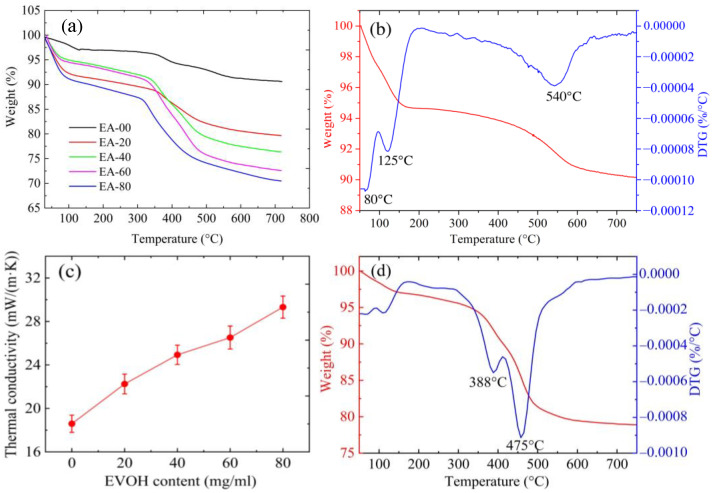
(**a**) Thermo-gravimetric analysis (TGA) curves of EVOH-modified aerogels. (**b**) TGA and DTG curves of the pure aerogel. (**c**) Thermal conductivity of EVOH-modified aerogels. **(d**) TGA and DTG curves of EA-20.

The thermal stability of both the EVOH-modified aerogels and pure aerogels was determined using TGA and DTG. As shown in Figure 6a, the thermal degradation of EVOH-modified aerogels occurred in three distinct stages. The first stage, ranging from 80 °C to 150 °C, is mainly attributed to the evaporation of adsorbed water and residual solvents. The second stage, occurring between 300 °C and 550 °C, is characterized by the rapid decomposition of EVOH, resulting in significant mass losses that vary with the EVOH concentration. Stabilization takes place at approximately 650 °C, with no further mass loss observed between 650 °C and 750 °C. The DTG curves in Figure 6d show two distinct mass-loss peaks, with the peak at 475 °C corresponding to the maximum rate of EVOH decomposition. In contrast, the DTG curve of pure aerogels in Figure 6b shows no mass loss in this range but instead exhibits a characteristic peak at 540 °C, attributed to the condensation and dehydration of silanol groups. Based on these observations, it is concluded that the thermal stability of the EVOH-modified aerogels fabricated via the TIPS method extends up to 350 °C, representing an improvement of approximately 70 °C compared to the PMMA-modified aerogels produced using the same approach [42].

The thermal conductivity of the EVOH-modified aerogels at ambient temperature was measured using a Hot Disk thermal constants analyzer (TPS 2500S). As illustrated in Figure 6c, the thermal conductivity of the aerogels exhibits a correlation with varying concentrations of EVOH. Notably, an increase in EVOH concentration enhances the bulk density, solid phase content, and average pore diameter of the aerogels, thereby augmenting the thermal conductivity. At an EVOH concentration of 80 mg/mL, the thermal conductivity peaks at 29.32 mW/(m·K). This increase in thermal conductivity can be attributed to the thickening of the connecting backbone, coupled with an increase in silica particle size.

### 2.5. Mechanical Properties

**Figure 7:**
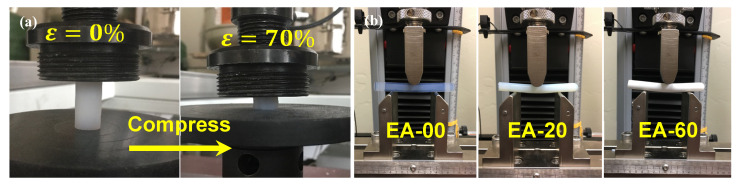

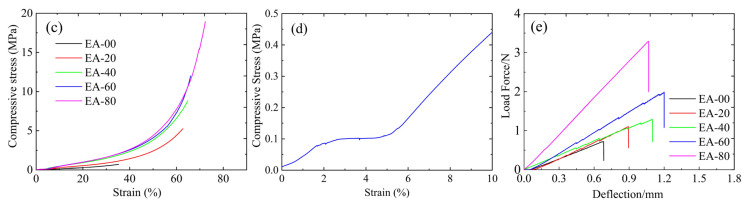
Mechanical properties of EVOH-modified aerogels. (**a**) images of the EA-80 aerogel before and after compression, (**b**) images of the EA-00, EA-20, and EA-60 aerogels under the three-point bending test, (**c**) compressive stress–strain curves of EVOH-modified aerogels, (**d**) stress–strain curve in the low strain range, and (**e**) flexural load–deflection curves of EVOH-modified aerogels.

The structural and mechanical properties of aerogels are influenced by various factors, including density, pore structure, and composition. In this study, the application of the thermally induced phase separation (TIPS) method enabled the uniform deposition of EVOH onto the silica particle network, leading to a significant enhancement in mechanical performance. The uniaxial compression test and three-point bending test were employed to characterize the mechanical behavior of the aerogels, as shown in Figure 7. The results obtained are summarized in Table 3, where the flexural strength, flexural modulus, compressive strength, and Young’s modulus are reported. The mechanical properties of the pure silica aerogels are similar to those previously reported for aerogels near this density [37,38,54]. In the case of the EA-80 aerogel, the flexural strength and modulus reached 0.545 MPa and 17.34 MPa, respectively, while the compressive strength and Young’s modulus were 24.8 and 5.7 times greater than those of the unmodified aerogel.

Figure 7 illustrates the mechanical performance of aerogels modified with varying EVOH concentrations. There is a noticeable enhancement in compressive and flexural strengths as the concentration of EVOH increases, indicating the significant reinforcement of EVOH on the network structure of aerogel. In the failure analysis results depicted in Figure 7c,d, the compression curves can be roughly divided into three stages based on their characteristic features. In the initial small strain region (<2%), a linear increase in stress with strain demonstrates the aerogel’s linear elasticity. Thereafter, the stress remains almost constant or increases slightly with strain (2–5%), representing the yield stage. Upon further compression (>5%), a rapid increase in stress leads to the fragmentation and collapse of the aerogel’s outer portion, transitioning into a densification stage where only a compacted core remains. Correspondingly, as the density of the aerogel increases, both the ultimate stress and the maximal strain rise. In the case of the EA-80 aerogel, the maximum compressive stress was 18.37 MPa, and the final amount of strain was 72.3%. The typical flexural load–deflection curves of aerogels are presented in Figure 7e. Similar to the compressive measurements, it is evident that the force and deflection of the EVOH-modified aerogel at final failure are higher than those of the unmodified aerogel. Specifically, the EA-80 sample exhibited failure at approximately 0.6 MPa of flexural strength, in contrast to the pure aerogel, which failed at about 0.1 MPa. Moreover, an increase in the EVOH concentration results in a notable decrease in flexural deformation under the same load. This indicates that the introduction of EVOH effectively enhances the ability of the modified aerogels to resist bending deformation.

**Figure 8:**
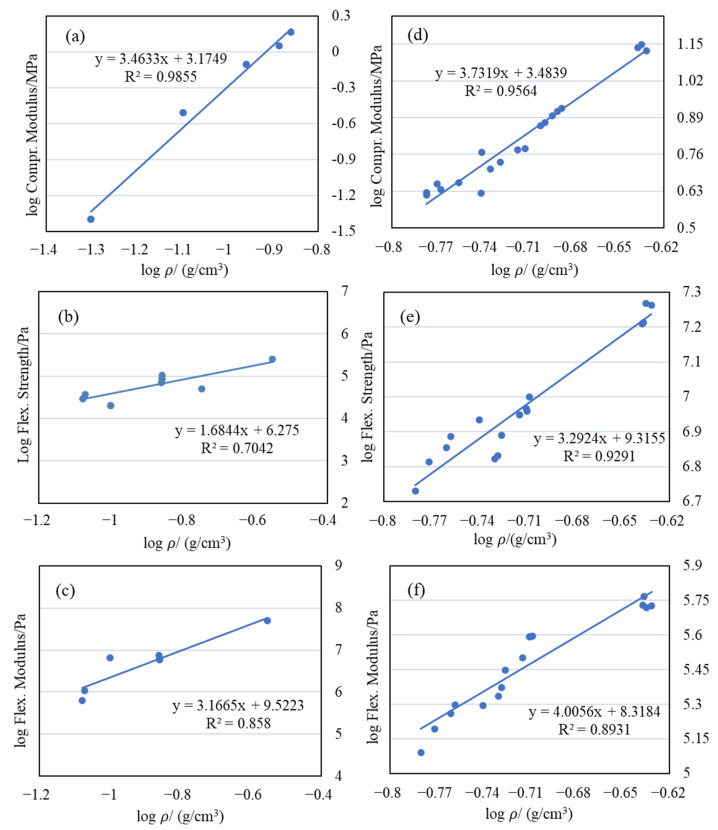
The log–log plots of mechanical strength and modulus versus density for EVOH-modified aerogels. (**a**–**c**) are for the pure aerogels; (**d**–**f**) are for the EVOH-modified aerogels.

As is typical for aerogels, there exists a power law relationship between the mechanical properties (strength and modulus) and the bulk density, which can be mathematically expressed as follows:

(1) $$E\propto \rho^{m}$$

(2) $$\sigma \propto \rho^{n}$$

where *E* is the Young’s modulus (MPa), σ is the strength (MPa) and ρ  is the bulk density of the aerogel (kg/m^3^). Figure 8 shows the log–log plots of compressive modulus, flexural strength, and flexural modulus versus bulk density for aerogels, with the experimental data fitting to a power law function, respectively. The main feature of these curves is the increase in compressive modulus, flexural modulus, and flexural strength with density. Additionally, for a given density, the EVOH-modified aerogels exhibit significantly higher mechanical strength and Young’s modulus than pure silica aerogels. The value of the exponent obtained by the best fitting of the data using Equations (1) and (2) was in the range of 3.0–4.0, which is in agreement with the prior findings for this class of materials [60,61,62,63,64].

**Figure 9:**
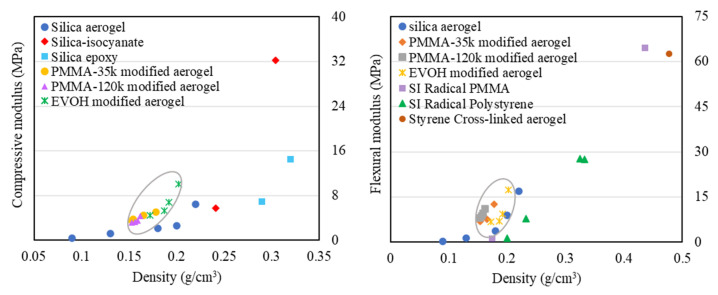
The comparison of the effect of polymer-modified aerogels via TIPS and other polymer-reinforced aerogels on mechanical properties [35,48,66,67].

Table 4 summarizes the slopes of the log–log plots of compressive modulus versus density for various aerogel systems. It is evident that the slopes of the log–log plots of compressive modulus versus density for polymer-modified aerogels synthesized via the TIPS method are noticeably higher than those reported for polyurethane composite silica aerogels, polyurethane aerogels, and cellulose composite silica aerogels. This result suggests that, for an equivalent increase in mass due to polymer incorporation, aerogels prepared using the TIPS method achieve a greater improvement in mechanical performance. Among the TIPS-modified systems, PMMA-modified aerogels exhibit even larger slopes than EVOH-modified ones, further demonstrating the versatility of the method. In addition, Figure 9 directly compares the compressive and flexural modulus of polymer-modified aerogels fabricated via TIPS with values reported for other polymer-reinforced aerogels in the literature. The results clearly demonstrate that the TIPS method not only preserves the characteristic low density and high porosity of silica aerogels but also provides superior reinforcement efficiency compared with conventional approaches such as fiber doping or polymer cross-linking. These findings highlight the potential of the TIPS strategy for fabricating mechanically robust, lightweight aerogels.

## 3. Conclusions

The focus of this study was the development of mechanically strong and lightweight EVOH-modified silica aerogels, fabricated via the thermally induced phase separation (TIPS) method and supercritical CO_2_ drying. Scanning electron microscopy analysis revealed that EVOH-modified aerogels possess a typical three-dimensional porous network structure with uniformly sized silica particles. Characterized by a homogeneous mesoporous structure and a robust skeleton network, the EVOH-modified silica aerogels exhibited low density (≤0.200 g/cm^3^), large specific surface area (≥401.0 m^2^/g), high porosity (86.8–91.6%), and low thermal conductivity (≤29.32 mW/(m·K)). More importantly, the incorporation of EVOH significantly enhanced mechanical performance, notably increasing the flexural strength to 0.545 MPa and compressive strength to 18.37 MPa, which is even greater than that of PMMA-modified aerogels in our previous study. This work highlights the efficacy of the TIPS method in enhancing the mechanical properties of silica aerogels through integration with poly(ethylene-co-vinyl alcohol), which provides a novel method for developing robust polymer-modified silica aerogels with less environmental burden.

## 4. Materials and Methods

### 4.1. Materials

Tetraethoxysilane (TEOS, 98%), isopropanol (IPA), and poly(ethylene-co-vinyl alcohol) (EVOH, 27 mol% ethylene content) were purchased from Sigma-Aldrich (St. Louis, MO, USA). Anhydrous ethanol (EtOH) was obtained from Decon Labs, Inc. (King of Prussia, PA, USA). Hydrochloric acid (HCl, 37%) was purchased from EMD Millipore Co. (Billerica, MA, USA). Ammonium hydroxide (NH_4_OH, 29%) was obtained from Kanto Co. (Portland, OR, USA). All reagents were of analytical grade and used as received without further purification.

### 4.2. Synthesis of EVOH-Modified Aerogels via TIPS

**Figure 10:**
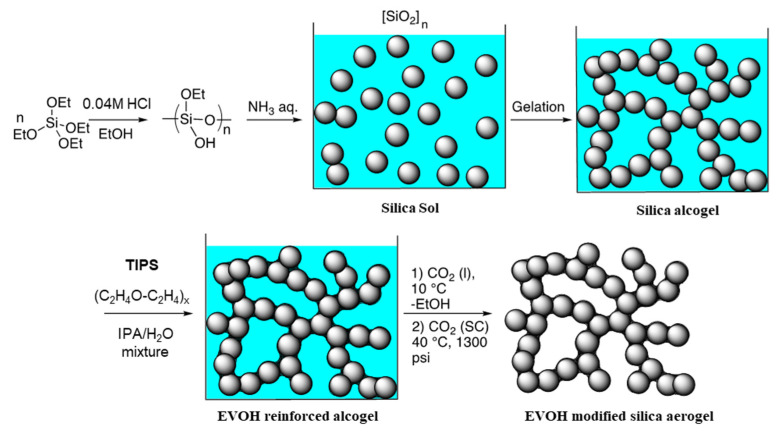
Schematic of the preparation process for EVOH-modified silica aerogels via TIPS.

Figure 10 illustrates the schematic procedure for preparing EVOH-modified silica aerogels via the TIPS method. Silica alcogels were synthesized through an acid–base catalyzed sol–gel polymerization process. Specifically, 20.8 g (0.1 mol) of TEOS, 18.4 g (0.4 mol) of ethanol, and 1.8 mL of 0.04 M hydrochloric acid solution were mixed to produce 47.4 mL of clear, colorless solution in a 100 mL flask. After magnetic stirring at 60 °C for 1.5 h, the solution was stored at −25 °C in the freezer until needed. 5 mL of the resulting hydrolyzed solution (0.0105 moles Si) and 1 mL ammonium hydroxide (0.25 mol/L, 0.056 moles H_2_O) was mixed and poured into a polypropylene vial, yielding a final silicate concentration of 1.75 mol/L. Transparent rigid gels formed within 17 min and were aged at room temperature for 12 h to strengthen the silica network. After aging, the gels were removed from the molds, immersed in IPA, sealed, and placed in an oven at 50 °C for 12 h.

The IPA within the gels was replaced by immersing each gel in 6 mL EVOH solution at 70 °C for 24 h to ensure complete infiltration. The EVOH solution was prepared by dissolving the polymer in a mixture of IPA and water (65 vol% IPA, 6 mL) at 70 °C under stirring until a homogeneous transparent solution was obtained. For the preparation of samples with varying EVOH concentrations, the following amounts of EVOH were dissolved in IPA/water (65 vol% IPA, 6 mL): for 20 mg/mL, 0.120 g (1.66 mmol); for 40 mg/mL, 0.240 g (3.33 mmol); for 60 mg/mL, 0.360 g (5.00 mmol); for 80 mg/mL, 0.480 g (6.67 mmol). Upon cooling the EVOH solutions to 10 °C, phase separation occurred, transforming the initially transparent monolithic silica gels into opaque white gels. The silica gels were maintained in the EVOH solutions at 10 °C for 24 h before being subjected to supercritical CO_2_ drying. The resulting EVOH-modified aerogels were designated as EA-20, EA-40, EA-60, and EA-80, corresponding to the concentration of the EVOH solution used. At least six samples were prepared for each formulation.

### 4.3. Characterization

Fourier transform infrared (FT-IR) spectroscopy was conducted by using a Nicolet FT-IR Spectrometer model Avatar 360 instrument in KBr pellets. Nitrogen adsorption/desorption isotherms were measured at 77K with a NOVA 4200e surface area and pore size analyzer (Quantachrome Instruments, Boynton Beach, FL, USA). Before the nitrogen adsorption experiments, all samples were degassed at 333K for at least 12 h under vacuum to remove residual solvent molecules. The specific surface area of the sample was calculated using the Brunauer–Emmett–Teller (BET) method at the linear part (0.05 < P/P_0_ < 0.25) of the adsorption branch. Scanning electron microscopy (SEM) analysis was conducted using a Nova Nanosem 450 instrument (FEI Co., Hillsboro, OR, USA) for the examination of aerogel morphology. Thermogravimetric analysis was performed using a TGA/DSC 3+ simultaneous thermal analyzer (Mettler Toledo, Zurich, Switzerland) under nitrogen and run at a temperature ramp rate of 10 °C/min. Thermal conductivities were determined at 20 °C from cylindrical specimens of monolithic aerogels using a Hot Disk TPS 2500S thermal (Hot Disk AB, Gothenburg, Sweden) conductivity meter. To estimate the porosity of the aerogels, the true density of the fabricated samples was measured using an TD2400 helium pycnometer (Builder Electronic, Beijing, China).

Compression tests were performed at 20 °C under 35% relative humidity using a CMT5305 universal testing machine (MTS Systems Co., LTD., Shanghai, China) equipped with a 5 kN load cell. In the absence of a dedicated standard for aerogel mechanical testing, the ASTM D695-23 [68] was employed in this work. Cylindrical specimens were polished with fine-grade (#1000) sandpaper on all surfaces and checked using an L-square to ensure the surfaces’ smoothness and parallelism. The final test specimens, with a slenderness ratio of 2:1, were compressed at a crosshead speed of 1 mm/min in accordance with the ASTM standard. Flexural properties were evaluated via a three-point flexural compression test using an Instron 5540 series single-column testing system with a 100 N load cell at a crosshead speed of 0.04 in/min, following ASTM D790-17 [69] and ASTM C1684-18 [70]. Samples were cylinders, approximately 74 mm in length by 8.5 mm in diameter. Reported strengths are the average values from the analyses of 6 samples.

## Figures and Tables

**Table 1:** Physical/structural properties of EVOH-modified aerogels.

Samples *	Bulk Density (g/cm^3^)	Surface Area (m^2^/g)	Porosity (%)	Linear Shrinkage (%)	EVOH Content TGA (g/g)	Mass Loss TGA (%)
EA-00	0.136 ± 0.002	874	93.7	8.6	0	9.0
EA-20	0.170 ± 0.004	618	91.6	8.2	0.09	21.1
EA-40	0.183 ± 0.005	568	89.5	7.6	0.145	23.6
EA-60	0.192 ± 0.001	429	89.1	7.3	0.152	27.4
EA-80	0.200 ± 0.001	401	86.8	6.9	0.176	29.5

* The samples are labeled as EA-X, where EA refers to the EVOH-silica aerogel, and X is the concentration of EVOH in the solution used in the TIPS, X mg/mL.

**Table 2:** Pore size analysis of EVOH-modified aerogels.

Samples	S_BET_ (m^2^/g)	S_NLDFT_ (m^2^/g)	S_DR_ (m^2^/g)	V_total_ (cm^3^/g)	V_pore,NLDFT_ (cm^3^/g)	V_pore,DR_ (cm^3^/g)	D_pore,BJH_ (nm)
EA-00	874	928	2352	5.055	4.713	0.870	17.73
EA-20	618	686	1833	4.019	3.442	0.651	17.35
EA-40	568	587	1487	3.753	2.987	0.475	17.49
EA-60	429	453	1196	2.943	2.528	0.425	17.88
EA-80	401	410	1049	2.742	2.176	0.406	17.33

S_BET_: multipoint BET surface area; V_pore,BJH_: BJH method cumulative volume; V_pore,NLDFT_: NLDFT method cumulative pore volume; D_pore,BJH_: BJH method pore diameter (desorption branch).

**Table 3:** Mechanical properties of EVOH-modified aerogels.

Samples	Density (g/cm^3^)	Flex. Strength (MPa)	Flex. Modulus (MPa)	Compr. Strength (MPa)	Compr. Modulus (MPa)
EA-00	0.136 ± 0.002	0.098 ± 0.020	5.61 ± 0.24	0.74 ± 0.17	1.78 ± 0.31
EA-20	0.172 ± 0.006	0.172 ± 0.033	6.68 ± 0.97	5.73 ± 0.62	4.44 ± 0.26
EA-40	0.184 ± 0.005	0.232 ± 0.036	7.09 ± 0.61	8.71 ± 0.57	5.34 ± 0.75
EA-60	0.190 ± 0.002	0.374 ± 0.037	9.31 ± 0.60	14.89 ± 0.76	6.83 ± 0.47
EA-80	0.201 ± 0.001	0.545 ± 0.027	17.34 ± 1.24	18.37 ± 0.93	10.07 ± 0.34

Data are the average of at least six stress–strain analyses for every sample formulation.

**Table 4:** The slopes of log compressive modulus versus log density [42,65].

Samples	Slopes of Log Compr. Modulus Versus Log Density
Silica aerogel	3.46
PU-silica composites	3.70
PU aerogels	3.62
Pectin–silica composites	3.82
Cellulose–silica composites	3.23
EVOH-modified aerogels	3.73
PMMA-35k-modified aerogels	4.49
PMMA-120k-modified aerogels	6.52

## Data Availability

The original contributions presented in the study are included in the article. Further inquiries can be directed to the corresponding author.
